# A Statistical-Physics Refinement of Soft Covering

**DOI:** 10.3390/e28060647

**Published:** 2026-06-08

**Authors:** Neri Merhav

**Affiliations:** The Viterbi Faculty of Electrical and Computer Engineering, Technion—Israel Institute of Technology, Technion City, Haifa 3200003, Israel; merhav@ee.technion.ac.il

**Keywords:** random coding, soft covering, channel resolvability, free energy, phase transitions, annealed free energy, Rényi entropy, statistical mechanics, guesswork, hypothesis testing

## Abstract

We study the channel output distribution induced by a random code of rate *R* from the perspective of statistical physics. The central object is the partition function $Z_{n}\left(\beta |C\right)=\sum_{y^{n}}{\left[P_{Y^{n}|C}\left(y^{n}\right)\right]}^{\beta }$, where $y^{n}$ is the channel output vector, C is the code, and β>0 plays the role of inverse temperature. More precisely, our focus is on the associated *annealed free energy*, $\psi \left(\beta ,R\right)=\lim_{n\to \infty }\frac{1}{n}\log E\left[Z_{n}\left(\beta |C\right)\right]$, where the expectation is with respect to the randomness of C. This quantity encodes the full Rényi spectrum of the output distribution. The single-letter formula derived for the annealed free energy decomposes into two branches, which reflect a “competition” between two populations of codewords. One is the *bulk branch*, $\psi_{b}\left(\beta ,R\right)$, which is driven by typical codewords, and the other one is the *sparse branch* $\psi_{s}\left(\beta ,R\right)$, which is driven by atypical codewords, where the qualifiers ‘typical’ and ‘atypical’ are in a sense that will become apparent later. We analyze the phase structure of each branch separately and characterize their competition. Both branches are derived for all β>0. The phase boundary $R^{🟉}\left(\beta \right)$, where the two branches are equal, is analyzed for β≥1, where it has an explicit closed-form expression. The phase diagram in the first quadrant of the (β,R) plane has four regions separated by three boundaries: $R=I^{b}\left(\beta \right)$ (bulk branch transition), $R=R^{🟉}\left(\beta \right)$ (bulk–sparse competition boundary), and $R=I^{s}\left(\beta \right)$ (sparse branch transition), all meeting at the point (β,R)=(1,I(X;Y)), where I(X;Y) is the mutual information induced by the input type and the channel. Applications to guesswork, channel resolvability, and hypothesis testing are discussed, and all the results are illustrated with a numerical example of a Z-channel.

## 1. Introduction

Given a codebook $C={\left\{x^{n}\left(m\right)\right\}}_{m=1}^{M}$ of size $M=e^{nR}$ and a discrete memoryless channel (DMC), {W(y|x)}, the induced channel output distribution is given by the mixture

(1) $$P_{Y^{n}}\left(y^{n}\right)=\frac{1}{M}\sum_{m=1}^{M}W^{n}\left(y^{n}|x^{n}\left(m\right)\right).$$

This object is central to channel resolvability [1] and the soft-covering problem (see, e.g., [2,3,4] and the references therein). Classical results, in this context, focus on a single threshold: if R>I(X;Y), I(X;Y) is the mutual information induced by the input distribution and the channel *W*, then the distribution $P_{Y^{n}}$ converges to the i.i.d. product law $P_{Y}^{⊗n}$ in total variation as well as in some other metrics between probability distributions.

This description, however, captures average behavior only. It says nothing about the internal geometry of $P_{Y^{n}}$: how the probability mass is distributed across output sequences, how many codewords support a typical and an atypical output, or how the self-information, $-\frac{1}{n}\log P_{Y^{n}}\left(y^{n}\right)$, fluctuates. These questions require a deeper journey beyond the Shannon entropy and mutual information.

In this work, we study $P_{Y^{n}}$ through the function

(2) $$Z_{n}\left(\beta \right)=\sum_{y^{n}}{\left[P_{Y^{n}}\left(y^{n}\right)\right]}^{\beta }=\sum_{y^{n}}\exp\left\{-\beta \log\left[1/P_{Y^{n}}\left(y^{n}\right)\right]\right\},\;\beta >0,$$

where the second representation is readily recognized as a *partition function*, with β>0 playing the role of *inverse temperature* and $\log\left[1/P_{Y^{n}}\left(y^{n}\right)\right]=-\log P_{Y^{n}}\left(y^{n}\right)$ being the *energy function* (a.k.a. the *Hamiltonian*) associated with every *micro-state* $y^{n}$. In other words, this is identified as the canonical partition function of a statistical-mechanics system whose micro-states are channel output sequences. Two special values bracket the range: $Z_{n}\left(1\right)=1$ (normalization, β=1) and $\lim_{\beta \to \infty }{\left[Z_{n}\left(\beta \right)\right]}^{1/\beta }=\max_{y^{n}}P_{Y^{n}}\left(y^{n}\right)$ (the mode, or the ground state in physics jargon). In general, $\frac{1}{1-\beta }\log Z_{n}\left(\beta \right)$ is exactly the definition of $H_{\beta }\left(P_{Y^{n}}\right)$, the Rényi entropy of order β≠1, pertaining to the output distribution $P_{Y^{n}}$, which is non-increasing in β. The associated *free energy* ψ(β,R) encodes the exponential growth/decay rate of $Z_{n}\left(\beta \right)$ as a function of *n*. Since $Z_{n}\left(\beta \right)$ depends on the random codebook and, actually, should be denoted $Z_{n}\left(\beta |C\right)$, we study the free energy behavior for the average code, which is identified with the *annealed free energy*,

(3) $$\psi \left(\beta ,R\right)=\lim_{n\to \infty }\frac{1}{n}\log E\left\{Z_{n}\left(\beta |C\right)\right\},$$

where the existence of the limit will be evident from the exponentially tight analysis in the proof of Theorem 1 in the sequel. Here E{·} denotes the expectation operator with respect to (w.r.t.) the randomness of the code C. Its phase structure—the rich dependence on β and *R*—is the main subject of this work.

It should be emphasized that many earlier statistical-mechanics analyses of random coding were based on Derrida’s random energy model (REM) (see, e.g., [5,6] and the references therein). These are based on partition functions whose micro-states were the channel inputs (i.e., the codewords) for a fixed channel output sequence $y^{n}$, that is,

(4) $$Z_{n}^{\mathrm{input}}\left(\beta \right)=\sum_{m=1}^{M}{\left[W^{n}\left(y^{n}|x^{n}\left(m\right)\right)\right]}^{\beta },$$

for fixed $y^{n}$, whereas, here, the micro-states are the channel outputs, as mentioned earlier. This difference is significant because, here, each energy term, $-\log P_{Y^{n}}\left(y^{n}\right)$, is itself a log-partition function over codewords. This two-level structure makes the problem considerably harder and richer than that of an ordinary REM.

The main contributions of this work are as follows. We derive the annealed free energy and analyze the phase structure of its two branches (Theorems 1 and 3). The free energy decomposes into a *bulk branch* and a *sparse branch*, reflecting a competition between two populations of codewords. The bulk branch is driven by *typical* codewords and the sparse branch is driven by *atypical* codewords, where the meanings of the qualifiers ‘typical’ and ‘atypical’ will become apparent in the sequel. Both branches are derived for all β>0 and analyzed separately. The bulk branch, $\psi_{b}\left(\beta ,R\right)$ (Section 3), has a single phase transition at a critical rate $R=I^{b}\left(\beta \right)$—the mutual information of the bulk optimizer (the unconstrained optimizer of the bulk branch)—where a rate constraint changes from inactive to active. A similar comment applies to the sparse branch, $\psi_{s}\left(\beta ,R\right)$, whose phase boundary is $R=I^{s}\left(\beta \right)$. The total annealed free energy ψ(β,R) has a phase boundary $R^{🟉}\left(\beta \right)$ given by an explicit closed-form formula (Theorem 3(b), Section 3). Together, the three boundaries divide the (β,R) quadrant {R≥0,β≥1} into four regions, denoted A, B, C, and D. By construction, $\psi \left(\beta ,R\right)\geq \psi_{b}\left(\beta ,R\right)$ always, with equality in regions A and B and strict inequality in regions C and D, where the sparse branch dominates. For the sparse branch, a fully explicit closed-form formula, in terms of channel transition probabilities and β alone, holds whenever the rate is below $I^{s}\left(\beta \right)$ (see Equation (29)). All the results are illustrated in a numerical example in Section 4.

A few words are in order with regard to earlier related work and the differences relative to the present work. The model (1) is the canonical object of channel resolvability and the soft-covering problem. Han and Verdú [1] established the resolvability threshold R=I(X;Y) under total variation, $∥P_{Y^{n}}-P_{Y}^{⊗n}{\;∥}_{\mathrm{TV}}\to 0$ for R>I(X;Y), and showed the threshold is tight. Hayashi [3] derived exponential convergence rates under total variation and normalized relative entropy, a.k.a. the Kullback–Leibler (KL) divergence. Hou and Kramer [4] extended the analysis to the Rényi divergence $D_{\alpha }\left(P_{Y^{n}}∥P_{Y}^{⊗n}\right)$, showing the critical rate remains I(X;Y) for all α∈(0,∞) but the exponents differ. Yu and Tan [7] characterized the *Rényi resolvability*—the minimum rate *R* required for the Rényi divergence $D_{\alpha }\left(P_{Y^{n}|C}∥P_{Y}^{⊗n}\right)$ to vanish asymptotically. They showed that, for α≤1, the threshold remains I(X;Y), while, for α>1, it is strictly larger than I(X;Y) and depends on α.

Our work is complementary to [7]: rather than characterizing the *threshold rate* at which a divergence to $P_{Y}^{⊗n}$ vanishes, we study the exact value of the annealed free energy at every rate *R* and inverse temperature β, revealing a two-branch phase diagram with three distinct phase boundaries. We analyze the *below-threshold* regime with the same precision as the above-threshold regime and expose qualitative phenomena—condensation, sparse-branch dominance, and the annealed/quenched gap—invisible to divergence-based analyses. In particular, our bulk branch boundary, $R=I^{b}\left(\beta \right)$, for β>1 is precisely the Rényi resolvability threshold of [7], now seen as one of three phase boundaries in a richer thermodynamic landscape.

In general, all the earlier results, in this context, share a common feature: they measure how close $P_{Y^{n}}$ is to a *target* distribution $P_{Y}^{⊗n}$ via some metric, and they all identify R=I(X;Y) as the single relevant threshold. The present paper asks a fundamentally different and complementary question: not how close $P_{Y^{n}}$ is to a target but what is the *internal geometry* of $P_{Y^{n}}$ itself?

Our partition function $Z_{n}\left(\beta \right)$ encodes the Rényi structure of $P_{Y^{n}}$ without reference to any external target distribution and reveals phenomena invisible to total variation or KL divergence. It turns out that the classical threshold, R=I(X;Y), is only the *beginning* of the story: the (β,R) quadrant {β≥1,R≥0} is divided into *four* distinct regions by three phase boundaries, all passing through the point (β,R)=(1,I(X;Y)). Concretely:

- Even for R>I(X;Y) (where $∥P_{Y^{n}}-P_{Y}^{⊗n}{∥}_{\mathrm{TV}}\;\to 0$), the annealed free energy reveals a *condensed phase*: when $R<I^{b}\left(\beta \right)$ for some β>1, the bulk branch of ψ has an active rate constraint, indicating that probability mass concentrates on outputs supported by sub-exponentially few codewords—output condensation that is invisible to total variation distance.
- The annealed free energy has a phase boundary $R=R^{🟉}\left(\beta \right)$ where the sparse branch takes over from the bulk branch, signaling that the atypical codewords dominate the ensemble average.
- Since $\log Z_{n}\left(\beta |C\right)=\left(1-\beta \right)\;H_{\beta }\left(P_{Y^{n}|C}\right)$ exactly for every fixed codebook (by the definition of Rényi entropy), the bulk branch $\psi_{b}\left(\beta ,R\right)$ encodes the Rényi entropy rate $H_{\beta }\left(P_{Y^{n}|C}\right)/n$ of the output mixture for a typical codebook (for β≥1 and $R>R^{🟉}\left(\beta \right)$; this is proved in Theorem 2; see also Appendix A). The full annealed free energy thus encodes the complete Rényi-order profile of the output distribution, of which classical soft covering (β→1) is a single cross-section.

In summary, this work does not contradict soft-covering results but refines and extends them by revealing the fine structure of $P_{Y^{n}}$ that lies beneath the single threshold R=I(X;Y). These structural findings have direct operational consequences, which we now describe.

1. *Guesswork and Rényi entropy.* Arikan [8] showed that guesswork moments $E[G{\left(Y^{n}\right)}^{s}]$ grow as $e^{nsH_{1/(1+s)}\left(P_{Y^{n}}\right)}$. The annealed free energy encodes the Rényi entropy rate of the output mixture, extending Arıkan’s analysis to the case where the source is itself a random-coding mixture. The phase structure of ψ(β,R) produces regime changes in guessing complexity that are absent in the i.i.d. case.
2. *Hypothesis testing.* The Chernoff exponent [9] for testing $P_{Y^{n}|C}$ against $P_{Y}^{⊗n}$ equals $\xi \left(R\right)=\max_{0\leq \beta \leq 1}\left[\left(1-\beta \right)\log|Y|-\psi \left(\beta ,R\right)\right]$, directly connecting our free energy to the optimal test. With $P_{Y}$ being uniform, ξ(R)=0 for R≥I(X;Y), meaning the two distributions are not exponentially distinguishable at and above the soft-covering threshold. The phase structure of ψ(β,R) produces qualitative regime changes in ξ(R) as *R* varies, and it connects to the Rényi resolvability results of Yu and Tan [7]; see Section 5.
3. *Statistical mechanics of codes.* Sourlas [10] established the connection between linear codes and spin-glass models. Montanari [11] analyzed the phase transition in turbo codes. Mézard and Montanari [5] (Chapters 5 and 6) and Merhav [6] provided a comprehensive treatment of random coding via statistical physics, with the partition function summing over codewords (inputs) for a fixed received sequence. The present work complements [6] by placing the partition function over outputs: the resulting two-level hierarchical model has a distinct phase structure governed by the code rate *R* rather than by signal-to-noise ratio.

The outline of the remainder of this article is as follows. Section 2 defines the model and establishes the notation conventions. Section 3 derives the annealed free energy and analyzes the phase structure of its bulk and sparse branches; the phase boundary $R^{*}\left(\beta \right)$ has an explicit closed-form formula for β≥1. Section 4 presents the phase diagram (four regions, β≥1). Section 5 discusses applications and the implications of our results. Finally, Section 6 summarizes the paper and provides an outlook.

## 2. Model, Definitions and Notation

Throughout the paper, *n* denotes the block length. An *n*-vector over alphabet X is displayed as $x^{n}=\left(x_{1},x_{2},\ldots ,x_{n}\right)\in X^{n}$, X being the single-letter finite alphabet. Similarly, $y^{n}\in Y^{n}$, where the single-letter alphabet Y is also finite. The empirical distribution (type) of $x^{n}$ is denoted ${\hat{P}}_{x^{n}}\left(a\right)=\frac{1}{n}\#\left\{i:\;x_{i}=a\right\}$ for all a∈X. A similar definition applies to joint types of pairs of sequences, $\left(x^{n},y^{n}\right)$. A DMC *W* is a stochastic matrix W:X→Y, and, for sequences $x^{n}$ and $y^{n}$, we write $W^{n}\left(y^{n}|x^{n}\right)=\prod_{i=1}^{n}W\left(y_{i}|x_{i}\right)$. Throughout the sequel, all logarithms are natural (base *e*). Accordingly, information measures are given in nats. We use the standard exponential-equivalence notation f(n)≐g(n) to mean $\lim_{n\to \infty }\frac{1}{n}\log\frac{f(n)}{g(n)}=0$; i.e., *f* and *g* have the same exponential growth/decay rate. The notation of information measures is as follows. D(P∥Q) is the Kullback–Leibler (KL) divergence,

(5) $$D\left(P∥Q\right)=\sum_{x\in X}P\left(x\right)\log\frac{P(x)}{Q(x)}.$$

Throughout the paper, $Q_{XY}$ denotes a joint distribution on X × Y with *X*-marginal given by $Q_{X}=P_{X}$, $P_{X}$ being a fixed input distribution on the finite input alphabet X. When $Q_{XY}$ needs to appear as a *subscript* in an information measure, we abbreviate $Q_{XY}$ by *Q* to avoid cumbersome notation. Thus, $H_{Q}\left(Y\right)$, $H_{Q}\left(Y|X\right)$, and $I_{Q}\left(X;Y\right)$ stand for $H_{Q_{XY}}\left(Y\right)$, $H_{Q_{XY}}\left(Y|X\right)$, and $I_{Q_{XY}}\left(X;Y\right)$, respectively, which are the entropy of *Y*, the conditional entropy of *Y* given *X*, and the mutual information between *X* and *Y*, respectively, all induced by $Q_{XY}$. On the other hand, when $Q_{XY}$ appears as an argument of a certain functional, the full notation is used. The notation I(X;Y) (without subscript) refers to the mutual information induced by the fixed single-letter channel input distribution $P_{X}$ and the channel transition probability matrix *W*, i.e., $I\left(X;Y\right)=I_{P_{X}W}\left(X;Y\right)$. The KL divergence between a conditional distribution $Q_{Y|X}$ and *W*, with weighting by $P_{X}$, is defined by

(6) $$D(Q_{Y|X}∥W|P_{X})=\sum_{x\in X}P_{X}\left(x\right)\sum_{y\in Y}Q_{Y|X}\left(y|x\right)\log\frac{Q_{Y|X}\left(y|x\right)}{W(y|x)}.$$

A random codebook of rate R>0 and block length *n* is a collection $C={\left\{x^{n}\left(m\right)\right\}}_{m=1}^{M}$ and $M=\lfloor e^{nR}\rfloor$, where the codewords $x^{n}\left(m\right)$ are drawn independently and uniformly at random from the type class,

(7) $$T\left(P_{X}\right)=\left\{x^{n}\in X^{n}:{\hat{P}}_{x^{n}}\left(a\right)=P_{X}\left(a\right)\;\forall \;a\in X\right\},$$

the set of all *n*-sequences with empirical distribution exactly $P_{X}$. Given codebook C, the induced output distribution is

(8) $$P_{Y^{n}|C}\left(y^{n}\right)=\frac{1}{M}\sum_{m=1}^{M}W^{n}\left(y^{n}|x^{n}\left(m\right)\right).$$

For β>0, the partition function is defined as

(9) $$Z_{n}\left(\beta |C\right)=\sum_{y^{n}}{\left[P_{Y^{n}|C}\left(y^{n}\right)\right]}^{\beta }.$$

The *annealed free energy* is

(10) $$\psi \left(\beta ,R\right)=\lim_{n\to \infty }\frac{1}{n}\log E_{C}\left[Z_{n}\left(\beta |C\right)\right],$$

where the limit will be evident in the subsequent analysis. For a joint type $Q_{XY}$ on X×Y with marginal $Q_{X}=P_{X}$, define:

(11) $$\ell \left(Q_{XY}\right)=H_{Q}\left(Y|X\right)+D(Q_{Y|X}∥W|P_{X}).$$

Note that, for $\left(x^{n},y^{n}\right)$ of joint type $Q_{XY}$, $W^{n}\left(y^{n}|x^{n}\right)=e^{-n\ell (Q_{XY})}$. We shall often use the identity

(12) $$I_{Q}\left(X;Y\right)+\ell \left(Q_{XY}\right)=H_{Q}\left(Y\right)+D(Q_{Y|X}∥W|P_{X}).$$

Fix an output sequence $y^{n}$ of type $Q_{Y}$. The type-class enumerator $N(Q_{XY}|y^{n})$ counts how many codewords have a joint type (joint empirical distribution) $Q_{XY}$ with $y^{n}$:

(13) $$N\left(Q_{XY}|y^{n}\right)=\#\left\{m:\;\left(x^{n}\left(m\right),y^{n}\right)\in T\left(Q_{XY}\right)\right\},$$

where $T(Q_{XY})$ is the joint type class associated with the joint distribution $Q_{XY}$.

Using the method of types [12,13], it is readily observed that, given $y^{n}$, each randomly selected codeword, $x^{n}\left(m\right)$, satisfies $\left(x^{n}\left(m\right),y^{n}\right)\in T\left(Q_{XY}\right)$ with probability

(14) $$\frac{\left|T(Q_{X|Y}|y^{n})\right|}{\left|T(P_{X})\right|}≐\frac{e^{nH_{Q}\left(X|Y\right)}}{e^{nH_{Q}\left(X\right)}}=e^{-nI_{Q}\left(X;Y\right)},$$

where $T\left(Q_{X|Y}|y^{n}\right)=\left\{x^{n}:\;\left(x^{n},y^{n}\right)\in T\left(Q_{XY}\right)\right\}$ is the conditional type class, and where we have used the fact that $Q_{X}=P_{X}$. Since codewords are drawn independently at random from $T(P_{X})$, it is clear that $N(Q_{XY}|y^{n})$ is a binomial random variable with *M* trials and a probability of single success of the exponential order of $e^{-nI_{Q}\left(X;Y\right)}$.

## 3. Annealed Free Energy and Its Phase Structure

### 3.1. Statistical-Mechanics Perspective

Before presenting the formulas, we pause to explain the statistical-mechanics framework, why the qualifiers “annealed” and “quenched” are used, and why the analysis goes beyond a simple saddle-point calculation.

#### 3.1.1. Annealed vs. Quenched: Origin of the Terminology

The terms come from metallurgy and were adopted by the statistical-mechanics community to describe two ways of averaging over quenched (i.e., frozen) disorder. In a disordered system—such as a spin glass—the *disorder* (e.g., the random coupling constants) is fixed for each physical realization but is drawn from some distribution. *Annealing* is the physical process of heating a material and cooling it slowly so that the disorder equilibrates with the thermal fluctuations; mathematically, this corresponds to averaging the partition function *before* taking its logarithm as if the disorder itself participates in the thermal equilibrium:

$$\psi^{\mathrm{ann}}\left(\beta ,R\right)=\lim_{n\to \infty }\frac{1}{n}\log E_{C}\left[Z_{n}\left(\beta |C\right)\right].$$

*Quenching* is the opposite process: the disorder is frozen rapidly and does not equilibrate. The free energy of a typical frozen realization is then

$$\psi^{\mathrm{que}}\left(\beta ,R\right)=\lim_{n\to \infty }\frac{1}{n}E_{C}\left[\log Z_{n}\left(\beta |C\right)\right],$$

i.e., the logarithm is taken *before* averaging. In our setting, the “disorder” is the random codebook C; once drawn, it is fixed and does not change with β. The annealed free energy ψ(β,R) defined in (10) is $\psi^{\mathrm{ann}}\left(\beta ,R\right)$. By Jensen’s inequality (since log is concave), $\psi^{\mathrm{ann}}\left(\beta ,R\right)\geq \psi^{\mathrm{que}}\left(\beta ,R\right)$ always. The main subject of this paper is $\psi^{\mathrm{ann}}\left(\beta ,R\right)$ (Theorems 1 and 3 below); whether and when $\psi^{\mathrm{ann}}=\psi^{\mathrm{que}}$ is addressed in Theorem 2.

#### 3.1.2. The Annealed Free Energy and Its Information-Theoretic Meaning

The partition function $Z_{n}\left(\beta |C\right)=\sum_{y^{n}}{\left[P_{Y^{n}|C}\left(y^{n}\right)\right]}^{\beta }$ is defined in (9). Its exponential growth rate $\left(1/n\right)\log Z_{n}$ encodes how the output probability mass is distributed: large β emphasizes the most probable outputs (low energy, high $P_{Y^{n}|C}$), while β→0 weights all outputs equally. The special value β=1 gives $Z_{n}\left(1|C\right)=1$ (normalization), and, as shown below, $\left(1/n\right)\log Z_{n}\left(\beta |C\right)$ is proportional to the Rényi entropy $H_{\beta }\left(P_{Y^{n}|C}\right)$ of the output mixture (defined in the sequel). The annealed free energy $\psi \left(\beta ,R\right)=\lim_{n}\frac{1}{n}\log E\left[Z_{n}\left(\beta |C\right)\right]$ thus encodes the Rényi entropy profile of the output distribution averaged over codebooks. Its exact single-letter formula—derived in Theorem 1—reveals a competition between two populations of codewords, called the *bulk branch* $\psi_{b}\left(\beta ,R\right)$ and the *sparse branch* $\psi_{s}\left(\beta ,R\right)$, to be defined precisely in the sequel.

#### 3.1.3. Why This Is More than a Saddle-Point Calculation

A naive approach would replace $P_{Y^{n}|C}\left(y^{n}\right)$ by its expectation $E[P_{Y^{n}|C}\left(y^{n}\right)]$ and compute $Z_{n}$ deterministically. This is correct only if $Z_{n}$ concentrates around its mean at the exponential scale, i.e., if $\left(1/n\right)\log Z_{n}\approx \left(1/n\right)\log E\left[Z_{n}\right]$ with high probability. When this *self-averaging* property holds, the annealed and quenched free energies coincide and a saddle-point over the output type $Q_{Y}$ gives the answer. However, self-averaging can fail: the annealed average $E[Z_{n}\left(\beta |C\right)]$ may be dominated by a sub-exponential fraction of codebooks that are far from typical, specifically those containing a codeword whose joint type $Q_{XY}$ with some output $y^{n}$ satisfies $I_{Q}\left(X;Y\right)>R$ (a “sparse-type” codeword, in the terminology of the sequel). These atypical codebooks contribute negligibly in probability but dominate the expectation of $Z_{n}$, producing an annealed free energy strictly larger than the quenched one. Identifying when this happens—and characterizing both the typical behavior $\psi_{b}$ and the atypical behavior $\psi_{s}$ as a function of (β,R)—requires tracking two competing saddle points simultaneously rather than a single one. This competition produces the *two-branch* structure of ψ(β,R) and the three phase boundaries (to be derived in Theorem 3).

#### 3.1.4. Phase Transitions and the Two-Level Hierarchical Structure

The phase boundaries of ψ(β,R)—to be characterized explicitly in Theorem 3 and illustrated in Section 4—signal qualitative changes in which configurations dominate $Z_{n}$. This is qualitatively different from earlier statistical-mechanics analyses of random codes (see [5,6]), which placed the partition function over *codewords* (inputs) for a fixed output $y^{n}$: $Z_{n}^{\mathrm{input}}\left(\beta \right)=\sum_{m=1}^{M}{\left[W^{n}\left(y^{n}|x^{n}\left(m\right)\right)\right]}^{\beta }$, as in (4). In that case, the energies $-\log W^{n}\left(y^{n}|x^{n}\left(m\right)\right)$ are approximately i.i.d. across codewords (analogous to Derrida’s random energy model), and the analysis reduces to a single saddle point. In the present work, the energy of each output micro-state $y^{n}$ is $-\log P_{Y^{n}|C}\left(y^{n}\right)$, which is itself a log-partition function over codewords—a two-level hierarchical structure. This makes the energies neither independent nor identically distributed across output sequences $y^{n}$ and is the source of the richer two-branch phase diagram with three distinct boundaries.

### 3.2. Main Result: Formula for the Annealed Free Energy

In this section, we derive a single-letter expression for the annealed free energy and investigate its phase structure in the (β,R) plane. The first main result is the following.

**Theorem** **1** (Annealed Free Energy, β>0)**.** *For all β>0:*

(15) $$\psi \left(\beta ,R\right)=\max\;\left\{\psi_{b}\left(\beta ,R\right),\;\psi_{s}\left(\beta ,R\right)\right\},$$

*with*

(16) $$\begin{array}{cc} \psi_{b}\left(\beta ,R\right) & =\underset{\begin{array}{c} Q_{XY}:\;Q_{X}=P_{X}\\ I_{Q}\left(X;Y\right)\leq R \end{array}}{\sup}\left\{H_{Q}\left(Y\right)-\beta \left[I_{Q}\left(X;Y\right)+\ell \left(Q_{XY}\right)\right]\right\}, \end{array}$$

(17) $$\begin{array}{cc} \psi_{s}\left(\beta ,R\right) & =R\left(1-\beta \right)+\underset{\begin{array}{c} Q_{XY}:\;Q_{X}=P_{X}\\ I_{Q}\left(X;Y\right)>R \end{array}}{\sup}\left\{H_{Q}\left(Y|X\right)-\beta \ell \left(Q_{XY}\right)\right\}. \end{array}$$

For convenience, we define the functional

(18) $$F\left(Q_{XY}\right):=H_{Q}\left(Y|X\right)-\beta \ell \left(Q_{XY}\right),$$

which is the common building block of both branches. Using the identity $H_{Q}\left(Y\right)=I_{Q}\left(X;Y\right)+H_{Q}\left(Y|X\right)$, we may present the two branches of the annealed free energy as

(19) $$\begin{array}{cc} \psi_{b}\left(\beta ,R\right) & =\underset{\begin{array}{c} Q_{XY}:\;Q_{X}=P_{X}\\ I_{Q}\left(X;Y\right)\leq R \end{array}}{\sup}\left[\left(1-\beta \right)I_{Q}\left(X;Y\right)+F\left(Q_{XY}\right)\right] \end{array}$$

(20) $$\begin{array}{cc} \psi_{s}\left(\beta ,R\right) & =R\left(1-\beta \right)+\underset{\begin{array}{c} Q_{XY}:\;Q_{X}=P_{X}\\ I_{Q}\left(X;Y\right)>R \end{array}}{\sup}F\left(Q_{XY}\right). \end{array}$$

**Proof of Theorem 1.**  We begin with the trivial identity,

(21) $$E\left[Z_{n}\left(\beta \right)\right]=E\left[\sum_{y^{n}}{\left[P_{Y^{n}|C}\left(y^{n}\right)\right]}^{\beta }\right]=\sum_{y^{n}}E\left\{{\left[P_{Y^{n}|C}\left(y^{n}\right)\right]}^{\beta }\right\}.$$

We next focus on $E[P_{Y^{n}|C}{\left(y^{n}\right)}^{\beta }]$ for a fixed $y^{n}$. Define $S\left(y^{n}\right)=\sum_{m=1}^{M}W^{n}\left(y^{n}|x^{n}\left(m\right)\right)$ so that $P_{Y^{n}|C}\left(y^{n}\right)=S\left(y^{n}\right)/M$. Since $M=e^{nR}$ is deterministic,

(22) $$E\;\left[P_{Y^{n}|C}{\left(y^{n}\right)}^{\beta }\right]=M^{-\beta }E\left\{{\left[S\left(y^{n}\right)\right]}^{\beta }\right\}=e^{-n\beta R}E\left\{{\left[S\left(y^{n}\right)\right]}^{\beta }\right\}.$$

The problem reduces to computing $E[S{\left(y^{n}\right)}^{\beta }]$. For each $y^{n}$,

(23) $$\begin{array}{ccc} E\{{\left[S\left(y^{n}\right)\right]}^{\beta }\} & = & E{\left[\sum_{\begin{array}{c} Q_{XY}:\;Q_{X}=P_{X}\\ Q_{Y}={\hat{P}}_{y^{n}} \end{array}}N\left(Q_{XY}|y^{n}\right)\cdot e^{-n\ell (Q_{XY})}\right]}^{\beta }\\ & ≐ & E{\left[\max_{\begin{array}{c} Q_{XY}:\;Q_{X}=P_{X}\\ Q_{Y}={\hat{P}}_{y^{n}} \end{array}}N\left(Q_{XY}|y^{n}\right)\cdot e^{-n\ell (Q_{XY})}\right]}^{\beta }\\ & = & E\left[\max_{\begin{array}{c} Q_{XY}:\;Q_{X}=P_{X}\\ Q_{Y}={\hat{P}}_{y^{n}} \end{array}}{\left[N\left(Q_{XY}|y^{n}\right)\right]}^{\beta }\cdot e^{-n\beta \ell (Q_{XY})}\right]\\ & ≐ & E\left[\sum_{\begin{array}{c} Q_{XY}:\;Q_{X}=P_{X}\\ Q_{Y}={\hat{P}}_{y^{n}} \end{array}}{\left[N\left(Q_{XY}|y^{n}\right)\right]}^{\beta }\cdot e^{-n\beta \ell (Q_{XY})}\right]\\ & = & \sum_{\begin{array}{c} Q_{XY}:\;Q_{X}=P_{X}\\ Q_{Y}={\hat{P}}_{y^{n}} \end{array}}E\left\{{\left[N\left(Q_{XY}|y^{n}\right)\right]}^{\beta }\right\}\cdot e^{-n\beta \ell (Q_{XY})}\\ & ≐ & \max_{\begin{array}{c} Q_{XY}:\;Q_{X}=P_{X}\\ Q_{Y}={\hat{P}}_{y^{n}} \end{array}}E\left\{{\left[N\left(Q_{XY}|y^{n}\right)\right]}^{\beta }\right\}\cdot e^{-n\beta \ell (Q_{XY})}, \end{array}$$

where the dotted equalities hold since the sum contains at most poly(n) non-negative terms. Now, the evaluation of $E{\left\{N\left(Q_{XY}|y^{n}\right)\right]}^{\beta }\}$ requires the following lemma, which is Theorem 4.2 of [14], and the proof is therein.

**Lemma 1**  (Moments of Binomial Enumerator)**.** *Let $N∼\mathrm{Binomial}(e^{nA},e^{-nB})$ with A,B>0 and β>0. Then,*

(24) $$E\left\{N^{\beta }\right\}≐\left\{\begin{array}{cc} e^{n\beta (A-B)} & A>B\\ e^{-n(B-A)} & A<B \end{array}\right.$$

The case A=B corresponds to a non-exponential behavior of $E\{N^{\beta }\}$, as can be seen by observing the limiting behavior of both cases. (As a side remark, when *N* is $\mathrm{Binomial}(e^{nA},\lambda e^{-nA})$ for some constant λ>0, then, in the limit of n→∞, *N* becomes a Poissonian random variable with parameter λ; that is, $Pr\left\{N=k\right\}\to \frac{\lambda^{k}e^{-\lambda }}{k!}$ for every non-negative integer *k*, and, therefore, the asymptotic βth moments are constants).

Lemma 1 is now used with the assignments A=R and $B=I_{Q}\left(X;Y\right)$. Accordingly, one must distinguish between types $\left\{Q_{XY}\right\}$ for which $R>I_{Q}\left(X;Y\right)$ as opposed to those with $R<I_{Q}\left(X;Y\right)$. Now,

(25) $$\begin{array}{ccc} E\{{\left[S\left(y^{n}\right)\right]}^{\beta }\} & ≐ & \max_{\begin{array}{c} Q_{XY}:\;Q_{X}=P_{X}\\ Q_{Y}={\hat{P}}_{y^{n}} \end{array}}E\left\{{\left[N\left(Q_{XY}|y^{n}\right)\right]}^{\beta }\right\}\cdot e^{-n\beta \ell (Q_{XY})}\\ & = & \max\{\max_{\begin{array}{c} Q_{XY}:\;Q_{X}=P_{X},\;Q_{Y}={\hat{P}}_{y^{n}}\\ I_{Q}\left(X;Y\right)\leq R \end{array}}E\left\{{\left[N\left(Q_{XY}|y^{n}\right)\right]}^{\beta }\right\}\cdot e^{-n\beta \ell (Q_{XY})},\\ & & \max_{\begin{array}{c} Q_{XY}:\;Q_{X}=P_{X},\;Q_{Y}={\hat{P}}_{y^{n}}\\ I_{Q}\left(X;Y\right)\geq R \end{array}}E\left\{{\left[N\left(Q_{XY}|y^{n}\right)\right]}^{\beta }\right\}\cdot e^{-n\beta \ell (Q_{XY})}\}\\ & ≐ & \max\{\max_{\begin{array}{c} Q_{XY}:\;Q_{X}=P_{X},\;Q_{Y}={\hat{P}}_{y^{n}}\\ I_{Q}\left(X;Y\right)\leq R \end{array}}e^{n\beta [R-I_{Q}\left(X;Y\right)]}\cdot e^{-n\beta \ell (Q_{XY})},\\ & & \max_{\begin{array}{c} Q_{XY}:\;Q_{X}=P_{X},\;Q_{Y}={\hat{P}}_{y^{n}}\\ I_{Q}\left(X;Y\right)\geq R \end{array}}e^{-n[I_{Q}\left(X;Y\right)-R]}\cdot e^{-n\beta \ell (Q_{XY})}\}. \end{array}$$

Since the latter expression involves maximization over $\left\{Q_{XY}\right\}$ when $Q_{Y}={\hat{P}}_{y^{n}}$ is held fixed, it is clear that it depends on $y^{n}$ only via ${\hat{P}}_{y^{n}}$. The number of $\left\{y^{n}\right\}$ with ${\hat{P}}_{y^{n}}=Q_{Y}$ is of the exponential order of $e^{nH_{Q}\left(Y\right)}$. Multiplying the per-$y^{n}$ contributions by $e^{nH_{Q}\left(Y\right)}$ and $e^{-n\beta R}$ yields the total contribution of type $Q_{Y}$, and, finally, summing over all $\left\{Q_{Y}\right\}$, which is exponentially equivalent to maximizing over all $\left\{Q_{Y}\right\}$, yields the asserted expression of ψ(β,R). The part pertaining to types with $I_{Q}\left(X;Y\right)\leq R$ is identified with $\psi_{b}\left(\beta ,R\right)$, and the one with $I_{Q}\left(X;Y\right)>R$ is associated with $\psi_{s}\left(\beta ,R\right)$. □

For future reference, we need the following definition.

**Definition** **1** (Sparse-feasible and bulk-feasible types)**.** *For a given rate R>0, a joint distribution $Q_{XY}$ with $Q_{X}=P_{X}$ is called:*

- Sparse-feasible *if $I_{Q}\left(X;Y\right)>R$; i.e., it satisfies the constraint of the sparse branch optimization;*
- Bulk-feasible *if $I_{Q}\left(X;Y\right)\leq R$; i.e., it satisfies the constraint of the bulk branch optimization. Every $Q_{XY}$ is either sparse-feasible or bulk-feasible (or both if $I_{Q}\left(X;Y\right)=R$)*.

### 3.3. The Sparse Branch: Formula and Phase Structure

The sparse branch of the annealed free energy,

(26) $$\psi_{s}\left(\beta ,R\right)=R\left(1-\beta \right)+\underset{\begin{array}{c} Q_{XY}:\;Q_{X}=P_{X}\\ I_{Q}\left(X;Y\right)>R \end{array}}{\sup}F\left(Q_{XY}\right),$$

captures the contribution of *sparse-type* codewords: those whose joint type $Q_{XY}$ with the output $y^{n}$ satisfies $I_{Q}\left(X;Y\right)>R$. For fixed $y^{n}$, such codewords are rarely encountered in a typical codebook of the ensemble.

**Definition** **2** (The annealed optimizer $Q_{\beta }^{s}$ and its mutual information $I^{s}\left(\beta \right)$)**.** *For each β>0, define*

(27) $$Q_{\beta }^{s}\left(y|x\right)=\frac{{\left[W\left(y|x\right)\right]}^{\beta }}{\sum_{y^{\prime }\in Y}{\left[W\left(y^{\prime }|x\right)\right]}^{\beta }}\;x\in X.$$

It can be readily shown that $Q_{\beta }^{s}$ maximizes $F\left(Q_{XY}\right)=H_{Q}\left(Y|X\right)-\beta \ell \left(Q_{XY}\right)$ over all $Q_{XY}$ with $Q_{X}=P_{X}$. Accordingly, define

(28) $$I^{s}\left(\beta \right):=I_{Q_{\beta }^{s}}\left(X;Y\right).$$

The curve $R=I^{s}\left(\beta \right)$ marks the point where $Q_{\beta }^{s}$ crosses from sparse-feasible ($I_{Q_{\beta }^{s}}\left(X;Y\right)>R$, so $Q_{\beta }^{s}$ optimizes the sparse branch) to bulk-feasible ($I_{Q_{\beta }^{s}}\left(X;Y\right)\leq R$, so $Q_{\beta }^{s}$ optimizes the bulk branch).

*Closed-form formula.* By Lemma 1, when $I_{Q}\left(X;Y\right)>R$, the βth moment of the enumerator satisfies $E\left\{{\left[N\left(Q_{XY}|y^{n}\right)\right]}^{\beta }\right\}≐e^{n[R-I_{Q}\left(X;Y\right)]}$ regardless of β. The resulting annealed calculation gives
  (29) $$\psi_{s}\left(\beta ,R\right)=R\left(1-\beta \right)+C\left(\beta \right)\;\mathrm{whenever}\;R<I^{s}\left(\beta \right),$$
  where
  (30) $$C\left(\beta \right)=\sum_{x\in X}P_{X}\left(x\right)\log\left(\sum_{y\in Y}{\left[W\left(y|x\right)\right]}^{\beta }\right).$$
  The formula ceases to hold when $R>I^{s}\left(\beta \right)$ as $Q_{\beta }^{s}$ is then bulk-feasible ($I_{Q_{\beta }^{s}}\left(X;Y\right)\leq R$) and hence does not comply with the rate constraint of the sparse branch.
*Phase structure.* The sparse branch has its own phase transition at $R=I^{s}\left(\beta \right)$:
  − For $R<I^{s}\left(\beta \right)$: $\psi_{s}\left(\beta ,R\right)=R\left(1-\beta \right)+C\left(\beta \right)$, the closed-form linear formula. The sparse branch is maximized by $Q_{\beta }^{s}$.
  − For $R>I^{s}\left(\beta \right)$: $Q_{\beta }^{s}$ is not sparse-feasible; the closed form R(1−β)+C(β) does not apply and the sparse branch must be evaluated directly from (26).
*Operational meaning.* The sparse branch governs the behavior of the ensemble average $E[Z_{n}\left(\beta |C\right)]$ when the latter is inflated by atypical codebooks. Specifically, when $\psi_{s}\left(\beta ,R\right)>\psi_{b}\left(\beta ,R\right)$ (which occurs for sufficiently small *R*, as characterized by the phase boundary $R^{🟉}\left(\beta \right)$ derived in Theorem 3 below), the annealed free energy satisfies $\psi \left(\beta ,R\right)=\psi_{s}\left(\beta ,R\right)$, meaning that $E[Z_{n}\left(\beta |C\right)]$ seems to be dominated by a sub-exponential fraction of codebooks with atypical codewords (those containing a sparse-type codeword with $I_{Q}\left(X;Y\right)>R$). A typical codebook has $\frac{1}{n}\log Z_{n}\left(\beta |C\right)\approx \psi_{b}\left(\beta ,R\right)$.

### 3.4. The Bulk Branch: Operational Meaning

We now explain why the bulk branch $\psi_{b}\left(\beta ,R\right)$ deserves a separate study within the analysis of ψ(β,R). As said before, the bulk branch, $\psi_{b}\left(\beta ,R\right)$, is the component of ψ(β,R) driven by *typical* codewords—those whose joint type with the output satisfies $I_{Q}\left(X;Y\right)\leq R$, i.e., codewords that are plausible given the code rate. A typical random codebook contains no sparse-type codewords (those with $I_{Q}\left(X;Y\right)>R$) with high probability, so, for a typical fixed codebook, $Z_{n}\left(\beta |C\right)≐e^{n\psi_{b}\left(\beta ,R\right)}$ at the exponential scale. The annealed free energy $\psi \left(\beta ,R\right)=\max\{\psi_{b}\left(\beta ,R\right),\psi_{s}\left(\beta ,R\right)\}$ can exceed $\psi_{b}\left(\beta ,R\right)$ (when the sparse branch dominates) because it is inflated by the rare codebooks that happen to contain a sparse-type codeword. In the language of statistical physics, $\psi_{b}\left(\beta ,R\right)$ is the *quenched* free energy, which is the free energy of a typical random code, while ψ(β,R) is the *annealed* free energy (the free energy of the disorder-averaged partition function). To support this observation, we now state the self-averaging property of $Z_{n}\left(\beta |C\right)$ for β≥1 and $R\geq R^{🟉}\left(\beta \right)$. The proof appears in the Appendix A.

**Theorem** **2** (Self-averaging and quenched free energy)**.** *For β≥1 and $R>R^{🟉}\left(\beta \right)$:*

*(i)*  $\lim_{n\to \infty }\frac{1}{n}\;E\left\{\log Z_{n}\left(\beta |C\right)\right\}=\psi_{b}\left(\beta ,R\right).$
*(ii)*  $\frac{1}{n}\log Z_{n}\left(\beta |C\right)\overset{\;a.s.\;}{\to }\psi_{b}\left(\beta ,R\right).$

The condition $R>R^{🟉}\left(\beta \right)$ is precisely the regime (regions A and B in the phase diagram) where the bulk branch dominates the annealed free energy: $\psi \left(\beta ,R\right)=\psi_{b}\left(\beta ,R\right)$ (Theorem 3). This is needed for the proof of the a.s. upper bound: Markov’s inequality on $E[Z_{n}]$ gives $\left(1/n\right)\log Z_{n}\leq \psi \left(\beta ,R\right)$ eventually a.s., and this equals $\psi_{b}\left(\beta ,R\right)$ only when $R>R^{🟉}\left(\beta \right)$. The lower bound of the proof holds under the weaker condition $R>I^{b}\left(\beta \right)$, so region C ($I^{b}\left(\beta \right)<R<R^{🟉}\left(\beta \right)$), where a typical codebook still lives in the bulk phase but the annealed average is inflated by rare sparse-type codebooks, remains open. Whether the result also extends to the condensed phase $R\leq I^{b}\left(\beta \right)$ or to β<1 likewise remains open.

By the definition of Rényi entropy, $H_{\beta }\left(Y\right)=\frac{1}{1-\beta }\log\sum_{y}P{\left(y\right)}^{\beta }$, so $\log Z_{n}\left(\beta |C\right)=\log\sum_{y^{n}}P_{Y^{n}|C}{\left(y^{n}\right)}^{\beta }=\left(1-\beta \right)\;H_{\beta }\left(P_{Y^{n}|C}\right)$ exactly for every codebook. Since $\frac{1}{n}\log Z_{n}\left(\beta |C\right)\to \psi_{b}\left(\beta ,R\right)$ a.s. (Theorem 2), the bulk branch encodes the Rényi entropy rate of the output mixture at order β:

(31) $$\psi_{b}\left(\beta ,R\right)≐\frac{1-\beta }{n}\;H_{\beta }\left(P_{Y^{n}|C}\right)$$

for a typical fixed codebook.

### 3.5. The Bulk Branch: Formula and Phase Structure

**Definition** **3** (The bulk optimizer $Q_{\beta }^{b}$ and its mutual information $I^{b}\left(\beta \right)$)**.** *For each β>0, define*

(32) $$Q_{\beta }^{b}={arg max}_{\begin{array}{c} Q_{XY}:\;Q_{X}=P_{X} \end{array}}\left[\left(1-\beta \right)I_{Q}\left(X;Y\right)+F\left(Q_{XY}\right)\right],$$

*the unconstrained maximizer of the bulk objective, and set*

(33) $$I^{b}\left(\beta \right):=I_{Q_{\beta }^{b}}\left(X;Y\right).$$

Obviously, for $R\geq I^{b}\left(\beta \right)$,

(34) $$\psi_{b}\left(\beta ,R\right)=\psi_{b,u}\left(\beta \right):=\underset{Q_{XY}:\;Q_{X}=P_{X}}{\sup}\left[\left(1-\beta \right)I_{Q}\left(X;Y\right)+F\left(Q_{XY}\right)\right].$$

The curve $R=I^{b}\left(\beta \right)$ is therefore the *bulk phase boundary*: for $R\geq I^{b}\left(\beta \right)$ the rate constraint $I_{Q}\left(X;Y\right)\leq R$ is inactive (bulk phase), while for $R<I^{b}\left(\beta \right)$ it is active (condensed phase).

The next lemma establishes an inequality relation between $I^{b}\left(\beta \right)$ and $I^{s}\left(\beta \right)$.

**Lemma** **2** (Ordering of the two optimizers)**.** *For β≥1:*

(35) $$I^{b}\left(\beta \right)\leq I^{s}\left(\beta \right),$$

*with strict inequality for β>1. At β=1, $I^{b}\left(1\right)=I^{s}\left(1\right)=I\left(X;Y\right)$*.

**Proof.**  By the optimality of $Q_{\beta }^{b}$ in the bulk objective:

(36) $$\begin{array}{ccc} F\left(Q_{\beta }^{b}\right)+\left(1-\beta \right)I^{b}\left(\beta \right) & \geq & F\left(Q_{\beta }^{s}\right)+\left(1-\beta \right)I^{s}\left(\beta \right)\\ & = & C\left(\beta \right)+\left(1-\beta \right)I^{s}\left(\beta \right), \end{array}$$

which, for β>1, is equivalent to

(37) $$I^{s}\left(\beta \right)-I^{b}\left(\beta \right)\geq \frac{C\left(\beta \right)-F\left(Q_{\beta }^{b}\right)}{\beta -1}$$

where the right-hand side is clearly non-negative since the denominator β−1 is positive, and the numerator is non-negative as C(β) is the global maximum of $F(Q_{XY})$, which cannot be smaller than $F(Q_{\beta }^{b})$. Therefore, $I^{s}\left(\beta \right)-I^{b}\left(\beta \right)$ is non-negative as well. □

### 3.6. Phase Structure of the Annealed Free Energy

The annealed free energy formula (Theorem 1) holds for all β>0. However, the analysis of the phase boundary between the bulk and sparse branches is restricted here to the range β≥1 for two reasons. The first reason is motivational: In the statistical-mechanics language, β≥1 is the low-temperature regime where $Z_{n}\left(\beta \right)$ is closely related to Rényi entropies of order at least one. This range captures the operationally most relevant quantities: β=1 (Shannon entropy and resolvability threshold), β=2 (collision entropy and birthday attacks), β→∞ (minimum entropy and $-\log\max_{y^{n}}P_{Y^{n}}\left(y^{n}\right)$), and general β>1 (guessing moments $E\left[G{\left(Y^{n}\right)}^{s}\right]≐e^{nsH_{1/(1+s)}}$ [8]. The second reason is that the range 0<β<1 does not appear to lend itself to closed-form analysis, the main issue being the lack of guarantee concerning the uniqueness of the solution *R* to the equation $\psi_{b}\left(\beta ,R\right)=\psi_{s}\left(\beta ,R\right)$, which is the phase boundary of the total annealed free energy, as will be defined next. In particular, for 0<β<1, the branches $\psi_{b}\left(\beta ,R\right)$ and $\psi_{s}\left(\beta ,R\right)$ may or may not cross, and existence and uniqueness of a crossing are not established in general.

**Definition** **4** (Annealed Phase Boundary)**.** *For β≥1, the* annealed phase boundary*$R^{🟉}\left(\beta \right)$ is the unique rate at which the bulk and sparse branches are equal:*

(38) $$\psi_{b}\left(\beta ,R^{🟉}\left(\beta \right)\right)=\psi_{s}\left(\beta ,R^{🟉}\left(\beta \right)\right).$$

Existence and uniqueness of $R^{🟉}\left(\beta \right)\in \left(I^{b}\left(\beta \right),I^{s}\left(\beta \right)\right)$ are established in Theorem 3(b) below, together with an explicit formula.

In order to keep track of the various phase boundaries and the quantities associated with them so far, the following table summarizes those ingredients (Table 1).

The following theorem provides a characterization of the phase structure of the annealed free energy.

**Theorem** **3.** *For any DMC W, any $P_{X}$, and any β≥1, R>0:*

*(a)*  *For $R\leq I^{s}\left(\beta \right)$, $Q_{\beta }^{s}$ is sparse-feasible and*
(39) $$\psi_{s}\left(\beta ,R\right)=R\left(1-\beta \right)+C\left(\beta \right).$$
*(b)*  *(Annealed phase boundary, β≥1.) Let*
(40) $$R^{🟉}\left(\beta \right):=\frac{C\left(\beta \right)-\psi_{b,u}\left(\beta \right)}{\beta -1},\;\beta >1,\;R^{🟉}\left(1\right):=I\left(X;Y\right).$$
*For all β≥1, $R^{🟉}\left(\beta \right)\in \left[I^{b}\left(\beta \right),I^{s}\left(\beta \right)\right]$, which is the unique rate at which $\psi_{s}\left(\beta ,R\right)=\psi_{b}\left(\beta ,R\right)$, and*
(41) $$\psi \left(\beta ,R\right)=\left\{\begin{array}{cc} \psi_{s}\left(\beta ,R\right) & R<R^{🟉}\left(\beta \right),\\ \psi_{b}\left(\beta ,R\right) & R\geq R^{🟉}\left(\beta \right). \end{array}\right.$$

The curve $R=R^{🟉}\left(\beta \right)$ is the *annealed phase boundary*.

**Proof.**  Part (a) was already shown earlier (but we include it here as part of the theorem for completeness since it is used in part (b)): Observe that, for $R\leq I^{s}\left(\beta \right)$, we have $I_{Q_{\beta }^{s}}\left(X;Y\right)\geq R$, so it is sparse-feasible. Since $Q_{\beta }^{s}$ achieves the global maximum, $F\left(Q_{\beta }^{s}\right)=\sup_{I_{Q}\left(X;Y\right)>R}F\left(Q_{XY}\right)=C\left(\beta \right)$, so $\psi_{s}\left(\beta ,R\right)=R\left(1-\beta \right)+C\left(\beta \right)$.

As for part (b), observe that, for $R\in (I^{b}\left(\beta \right),I^{s}\left(\beta \right))$, both simplified formulas hold simultaneously: $\psi_{b}\left(\beta ,R\right)=\psi_{b,u}\left(\beta \right)$ since $R>I^{b}\left(\beta \right)$) and $\psi_{s}\left(\beta ,R\right)=R\left(1-\beta \right)+C\left(\beta \right)$ (by part (a) since $R<I^{s}\left(\beta \right)$). Setting $\psi_{b}\left(\beta ,R\right)=\psi_{s}\left(\beta ,R\right)$ and solving gives immediately

(42) $$R^{🟉}\left(\beta \right)=\frac{C\left(\beta \right)-\psi_{b,u}\left(\beta \right)}{\beta -1}.$$

Substituting $\psi_{b,u}\left(\beta \right)=F\left(Q_{\beta }^{b}\right)+\left(1-\beta \right)I^{b}\left(\beta \right)$, we end up with:

(43) $$R^{🟉}\left(\beta \right)=I^{b}\left(\beta \right)+\frac{C\left(\beta \right)-F\left(Q_{\beta }^{b}\right)}{\beta -1}.$$

Since β−1>0 and $C\left(\beta \right)\geq F\left(Q_{\beta }^{b}\right)$, the second term is non-negative, giving $R^{🟉}\left(\beta \right)\geq I^{b}\left(\beta \right)$, with strict inequality since $F\left(Q_{\beta }^{b}\right)<C\left(\beta \right)$ strictly for β>1 (Lemma 2).

For the upper bound, recall from the proof of Lemma 2 that

(44) $$C\left(\beta \right)-F\left(Q_{\beta }^{b}\right)\leq \left(\beta -1\right)\left(I^{s}\left(\beta \right)-I^{b}\left(\beta \right)\right),$$

which gives

(45) $$\frac{C\left(\beta \right)-F\left(Q_{\beta }^{b}\right)}{\beta -1}\leq I^{s}\left(\beta \right)-I^{b}\left(\beta \right).$$

Substituting into (43): $R^{🟉}\left(\beta \right)\leq I^{s}\left(\beta \right)$, again with strict inequality for β>1. Hence $R^{🟉}\left(\beta \right)\in \left(I^{b}\left(\beta \right),I^{s}\left(\beta \right)\right)$, confirming the formula is valid.

To establish the uniqueness of the solution $R^{🟉}\left(\beta \right)$ to the equation $\psi_{b}\left(\beta ,R\right)=\psi_{s}\left(\beta ,R\right)$, it remains to rule out any additional solutions outside the interval $\left[I^{b}\left(\beta \right),I^{s}\left(\beta \right)\right]$. For $R\leq I^{b}\left(\beta \right)$, $Q_{\beta }^{s}$ is still sparse-feasible (since $R\leq I^{b}\left(\beta \right)<I^{s}\left(\beta \right)$), so part (a) gives $\psi_{s}\left(\beta ,R\right)=R\left(1-\beta \right)+C\left(\beta \right)$. The bulk constraint is active, so $\psi_{b}\left(\beta ,R\right)\leq \psi_{b,u}\left(\beta \right)$. Hence,

(46) $$\begin{array}{ccc} \Delta (\beta ,R) & := & \psi_{s}\left(\beta ,R\right)-\psi_{b}\left(\beta ,R\right)\\ & = & R\left(1-\beta \right)+C\left(\beta \right)-\psi_{b}\left(\beta ,R\right)\\ & \geq & R\left(1-\beta \right)+C\left(\beta \right)-\psi_{b,u}\left(\beta \right). \end{array}$$

The right side is positive at $R=I^{b}\left(\beta \right)$ (as shown above) and increasing as *R* decreases (since 1−β<0). Hence Δ(β,R)>0 for all $R\leq I^{b}\left(\beta \right)$. For $R\geq I^{s}\left(\beta \right)$, consider the following. Since $I^{b}\left(\beta \right)\leq I^{s}\left(\beta \right)\leq R$, the bulk constraint is inactive and $\psi_{b}\left(\beta ,R\right)=\psi_{b,u}\left(\beta \right)$. Since $R\geq I^{s}\left(\beta \right)>R^{🟉}\left(\beta \right)$ and $\psi_{b,u}\left(\beta \right)=R^{🟉}\left(\beta \right)\left(1-\beta \right)+C\left(\beta \right)$ (from the formula of $R^{🟉}\left(\beta \right)$), we have:

(47) $$\psi_{b}\left(\beta ,R\right)=\psi_{b,u}\left(\beta \right)=R^{🟉}\left(\beta \right)\left(1-\beta \right)+C\left(\beta \right)>R\left(1-\beta \right)+C\left(\beta \right)\geq \psi_{s}\left(\beta ,R\right),$$

where the strict inequality uses $R>R^{🟉}\left(\beta \right)$ and 1−β<0, and the last inequality is because $\psi_{s}\left(\beta ,R\right)=R\left(1-\beta \right)+\sup_{\left\{Q:\;Q_{X}=P_{X},\;I_{Q}\left(X;Y\right)>R\right\}}F\left(Q_{XY}\right)\leq R\left(1-\beta \right)+C\left(\beta \right)$. Hence Δ(β,R)<0 for all $R\geq I^{s}\left(\beta \right)$. □

## 4. A Numerical Example

In this section, we provide a numerical example and illustrate the behavior of the two branches of the annealed free energy as well as the phase boundary curves.

All phase diagrams are computed for an example of a Z-channel. The details are as follows. The input and output alphabets are X=Y={0,1}, and the crossover probability is p=0.45, i.e., W(0|0)=1, W(1|0)=0, W(0|1)=0.45, and W(1|1)=0.55. The input type is given by $P_{X}=\left(0.5,0.5\right)$. Accordingly, the resulting channel mutual information is I(X;Y)=0.2441.

**Figure 1:**
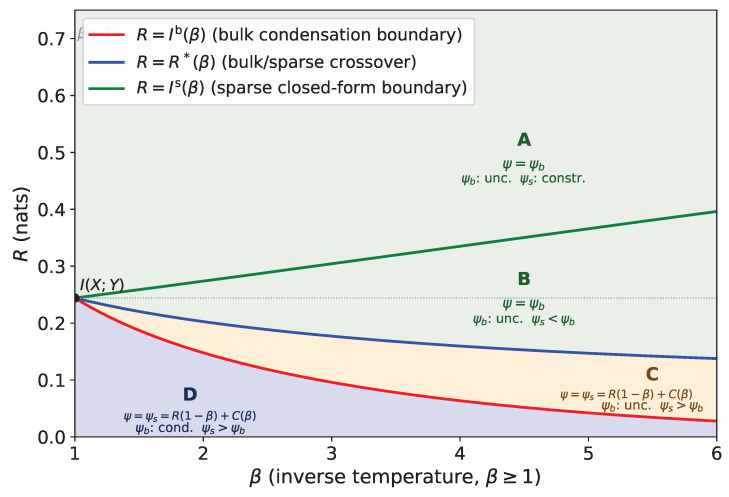
Phase boundaries for the Z-channel, β≥1. **Red:** $R=I^{b}\left(\beta \right)$, the bulk branch boundary (bulk rate constraint activates). **Blue:** $R=R^{🟉}\left(\beta \right)$, the annealed boundary where bulk = sparse (closed-form formula, Theorem 3(b)). **Green:**$R=I^{s}\left(\beta \right)$, the sparse branch boundary. All three meet at (β,R)=(1,I(X;Y)) (filled dot). Four regions (Remark 4): **A** ($R>I^{s}\left(\beta \right)$): $\psi_{b}$ unconstrained, $\psi_{s}$ constrained (sparse closed form does not apply); bulk dominates; **B** ($R^{🟉}\left(\beta \right)<R<I^{s}\left(\beta \right)$): bulk branch dominates ($\psi \left(\beta ,R\right)=\psi_{b}\left(\beta ,R\right)$); **C** ($I^{b}\left(\beta \right)<R<R^{🟉}\left(\beta \right)$): sparse branch dominates ($\psi \left(\beta ,R\right)=\psi_{s}\left(\beta ,R\right)$); **D** ($R<I^{b}\left(\beta \right)$): sparse branch dominates ($\psi \left(\beta ,R\right)=\psi_{s}\left(\beta ,R\right)$), bulk condensed.

Figure 1 plots all the phase boundaries together in the relevant quadrant of the (β,R) plane.

Three phase boundary curves are visible in Figure 1:

- $R=I^{b}\left(\beta \right)$ (red): the bulk branch boundary, where the unconstrained bulk optimizer crosses the rate constraint. At β=1: $I^{b}\left(1\right)=I\left(X;Y\right)$.
- $R=R^{🟉}\left(\beta \right)$ (blue): the annealed boundary where the bulk and sparse branches are equal, given by the closed-form Formula (40). At β=1: $R^{🟉}\left(1\right)=I\left(X;Y\right)$. Decreasing in β, staying above $I^{b}\left(\beta \right)$.
- $R=I^{s}\left(\beta \right)$ (green): the sparse branch boundary, where $Q_{\beta }^{s}$ (which maximizes $F\left(Q_{XY}\right)=H_{Q}\left(Y|X\right)-\beta \ell \left(Q_{XY}\right)$) has $I_{Q_{\beta }^{s}}\left(X;Y\right)=R$. For $R>I^{s}\left(\beta \right)$ the sparse closed form ceases to hold. At β=1: $I^{s}\left(1\right)=I\left(X;Y\right)$.

All three curves meet at the point (β,R)=(1,I(X;Y)), confirming that the soft-covering threshold is the unique point where all regions collapse to a single point.

For β≥1, the three phase boundaries divide the quadrant {β≥1,R>0} into four regions:

- **A** ($R>I^{s}\left(\beta \right)$, above all curves): $\psi_{b}\left(\beta ,R\right)$ is unconstrained ($I^{b}\left(\beta \right)<R$). $\psi_{s}\left(\beta ,R\right)$ is constrained: since $I^{s}\left(\beta \right)<R$, the sparse optimizer $Q_{\beta }^{s}$ is bulk-feasible and the sparse closed-form expression R(1−β)+C(β) does not apply. Bulk dominates: $\psi \left(\beta ,R\right)=\psi_{b}\left(\beta ,R\right)$ (since $R^{🟉}\left(\beta \right)<I^{s}\left(\beta \right)$).
- **B** ($R^{🟉}\left(\beta \right)<R<I^{s}\left(\beta \right)$): Bulk branch dominates: $\psi \left(\beta ,R\right)=\psi_{b}\left(\beta ,R\right)$. The bulk rate constraint is inactive ($R>I^{b}\left(\beta \right)$); no condensation.
- **C** ($I^{b}\left(\beta \right)<R<R^{🟉}\left(\beta \right)$): Sparse branch dominates: $\psi \left(\beta ,R\right)=\psi_{s}\left(\beta ,R\right)=R\left(1-\beta \right)+C\left(\beta \right)$ (closed-form linear formula). Rare-event codewords inflate the ensemble average; a typical codebook is in the bulk phase.
- **D** ($R<I^{b}\left(\beta \right)$, below the red curve): Sparse branch dominates: $\psi \left(\beta ,R\right)=\psi_{s}\left(\beta ,R\right)=R\left(1-\beta \right)+C\left(\beta \right)$. The bulk branch is condensed (rate constraint active; bulk optimizer constrained to $I_{Q}\left(X;Y\right)=R$), but the annealed free energy is inflated by rare sparse-type codebooks.

**Figure 2:**
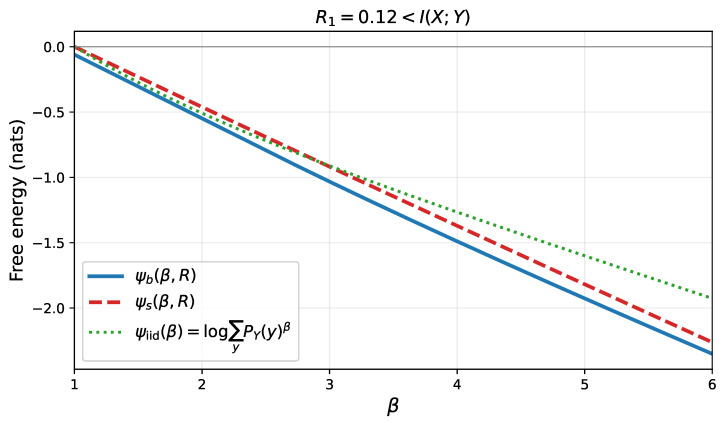
$R_{1}=0.12<I\left(X;Y\right)$. The sparse branch $\psi_{s}\left(\beta ,R_{1}\right)$ lies above the bulk branch for all β≥1, so the annealed free energy is dominated by the sparse branch; both lie well above the i.i.d. reference, reflecting ensemble inflation by rare codebooks.

**Figure 3:**
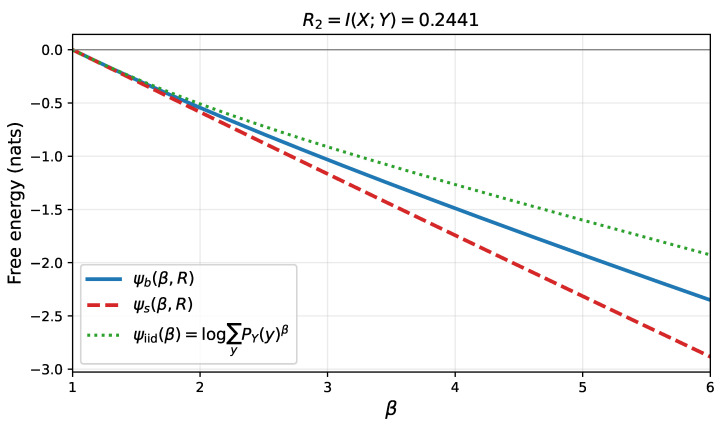
$R_{2}=I\left(X;Y\right)=0.2441$ nats (the soft-covering threshold). Both branches and the i.i.d. reference all vanish at β=1 (since $Z_{n}\left(1\right)=1$) and diverge differently for β>1.

**Figure 4:**
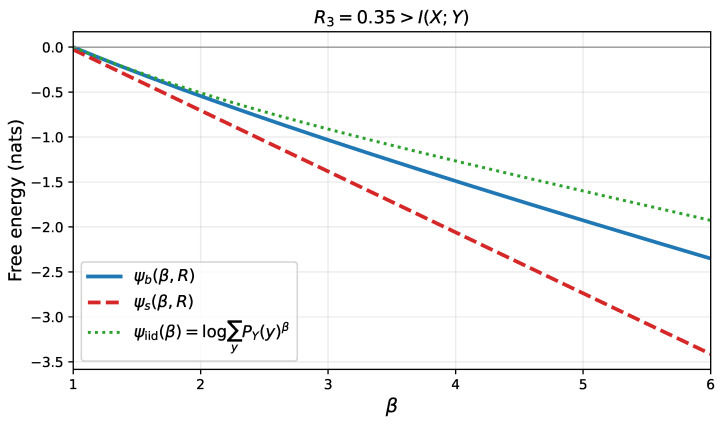
$R_{3}=0.35>I\left(X;Y\right)$. The bulk branch dominates throughout and tracks the i.i.d. reference closely, confirming that, above the soft-covering threshold, the output mixture approximates $P_{Y}^{⊗n}$ in the Rényi sense. In all three figures, $\psi_{\mathrm{iid}}\left(\beta \right)\leq \psi \left(\beta ,R\right)$, with strict inequality below I(X;Y): the random-coding mixture has strictly higher Rényi entropy than the i.i.d. output distribution at every order β≥1.

For the very same z-channel example, Figure 2, Figure 3 and Figure 4 display $\psi_{b}\left(\beta ,R\right)$ and $\psi_{s}\left(\beta ,R\right)$ as functions of β≥1 for three representative values of *R* (one below I(X;Y), another one equal to I(X;Y), and yet another one above I(X;Y)) and contrasts them with the *i.i.d. reference free energy*

(48) $$\psi_{\mathrm{iid}}\left(\beta \right):=\frac{1}{n}\log\left(\sum_{y^{n}}{\left[P_{Y}\left(y^{n}\right)\right]}^{\beta }\right)=\log\left(\sum_{y\in Y}{\left[P_{Y}\left(y\right)\right]}^{\beta }\right),$$

where $P_{Y}$ is the output distribution induced by $P_{X}$ and *W*. This is the free energy of the product distribution $P_{Y}^{⊗n}$, i.e., the distribution to which $P_{Y^{n}}$ converges for R>I(X;Y) based on well-known soft-covering results. Clearly, $\psi_{\mathrm{iid}}\left(\beta \right)$ is a purely smooth function with no phase transitions whatsoever.

A central message of this paper is that the classical soft-covering threshold R=I(X;Y) is far from the end of the story, and, as this numerical example illustrates, we refine and deepen those results in this work. As mentioned in the Introduction, classical results, like those of [1,3,7], tell us that $∥P_{Y^{n}}-P_{Y}^{⊗n}{∥}_{\mathrm{TV}}\to 0$ (and in other metrics) if and only if R>I(X;Y) and provide exponential rates for this convergence. Our statistical-physics analysis reveals that, even well above this threshold, $P_{Y^{n}}$ retains non-trivial internal structure that is invisible to total variation. Figure 2, Figure 3 and Figure 4 make this visible. The three plots of $\psi_{b}\left(\beta ,R\right)$, $\psi_{s}\left(\beta ,R\right)$, and the i.i.d. reference $\psi_{\mathrm{iid}}\left(\beta \right)=\log\left(\sum_{y}{\left[P_{Y}\left(y\right)\right]}^{\beta }\right)$ against β for $R_{1}<I\left(X;Y\right)=R_{2}<R_{3}$. At the classical threshold $R_{2}=I\left(X;Y\right)$ (second diagram), all three quantities vanish together at β=1 as required but diverge for β>1: the free energy $\psi (\beta ,R_{2})$ lies strictly above $\psi_{\mathrm{iid}}\left(\beta \right)$ for all β>1, meaning that $P_{Y^{n}}$ has strictly higher Rényi entropy than $P_{Y}^{⊗n}$ at every order. Above the threshold at $R_{3}>I\left(X;Y\right)$ (third diagram), the bulk branch closely tracks $\psi_{\mathrm{iid}}\left(\beta \right)$, confirming the soft-covering intuition that the output mixture approaches the i.i.d. law, but the gap, while small, remains nonzero at finite β, and quantifying it requires $\psi_{b}\left(\beta ,R_{3}\right)$. Below the threshold at $R_{1}<I\left(X;Y\right)$ (first diagram), the gap $\psi \left(\beta ,R_{1}\right)-\psi_{\mathrm{iid}}\left(\beta \right)$ is large and grows with β: the sparse branch dominates the ensemble average, and the random-coding output is far from i.i.d. in the Rényi sense even though TV-distance arguments do not apply here at all.

Specifically, $\psi_{b}\left(\beta ,R\right)$ is the Rényi entropy rate of $P_{Y^{n}}$ at order β for a typical codebook, and its phase transition at $R=I^{b}\left(\beta \right)$ implies a qualitative change in how probability mass is distributed over output sequences. In the *bulk phase* ($R>I^{b}\left(\beta \right)$), a typical output sequence is supported by exponentially many codewords. The output distribution is diffuse and “spread out” in a way that is consistent with the intuition behind soft covering. In the *condensed phase* ($R<I^{b}\left(\beta \right)$), a typical output appears to be supported by only sub-exponentially few codewords, meaning the mass of $P_{Y^{n}}$ concentrates on a sparse set of sequences even though the TV distance to $P_{Y}^{⊗n}$ may already be negligible. This condensation is visible in Figure 2: the first diagram ($R_{1}<I\left(X;Y\right)$) shows $\psi_{s}\left(\beta ,R_{1}\right)>\psi_{b}\left(\beta ,R_{1}\right)$ for all β≥1, so the ensemble average is dominated by the rare sparse-branch codebooks, while the third diagram ($R_{3}>I\left(X;Y\right)$) shows $\psi_{b}\left(\beta ,R_{3}\right)>\psi_{s}\left(\beta ,R_{3}\right)$, confirming typical-codebook dominance throughout.

The gap $\psi \left(\beta ,R\right)-\psi_{\mathrm{iid}}\left(\beta \right)\geq 0$ visible in all three diagrams has a direct operational meaning: it measures how much the random-coding output mixture exceeds the i.i.d. distribution in Rényi entropy at order β. Classical soft-covering results establish that this gap vanishes in total variation for R>I(X;Y). The figures show that, in Rényi entropy, the gap is nonzero for every β>1 and every finite *R*, decaying to zero only as R→∞.

## 5. Applications and Implications

In this section, we summarize several applications and implications of the results in this work. Further investigation of these applications is deferred to future work.

1. *Refined output statistics.* Resolvability shows $∥P_{Y^{n}}-{\left(P_{Y}\right)}^{n}{∥}_{\mathrm{TV}}\to 0$ for R>I(X;Y). Our phase diagram reveals that, even in this regime, $P_{Y^{n}}$ may be in the condensed phase (below $I^{b}\left(\beta \right)$ for large β), where mass appears to concentrate on outputs supported by a sub-exponential number of codewords. This is invisible to the total variation distance and other distances.
2. *Guesswork. *$E\left[G{\left(Y^{n}\right)}^{s}\right]≐e^{nsH_{1/(1+s)}\left(P_{Y^{n}}\right)}$ [8]. Since $H_{\beta }\left(P_{Y^{n}|C}\right)=\frac{1}{(1-\beta )n}\log Z_{n}\left(\beta |C\right)$ and $\frac{1}{n}\log Z_{n}\left(\beta |C\right)≐\psi \left(\beta ,R\right)$ (the full annealed free energy, including both bulk and sparse branches), the guessing exponent at β=1/(1+s) is $\frac{s}{n(1-\beta )}\log Z_{n}\left(\beta |C\right)≐\frac{s}{1-\beta }\psi \left(\beta ,R\right)$. The phase structure of ψ(β,R)—both its bulk phase transition at $R=I^{b}\left(\beta \right)$ and its bulk–sparse crossover at $R=R^{🟉}\left(\beta \right)$—therefore produces regime changes in guessing complexity absent in the i.i.d. case.
3. *Security.* In the condensed phase, even when $P_{Y^{n}}\approx {\left(P_{Y}\right)}^{n}$ in total variation, only a sub-exponential number of codewords may generate a typical output. A computationally powerful adversary can identify the message by examining which codewords are consistent with the observed output. Semantic security may therefore require operating strictly in the bulk phase for all β relevant to the adversary’s test.
4. *Hypothesis testing and the Chernoff exponent.* A natural question is how well one can distinguish the random-code output mixture $P_{Y^{n}|C}$ from the i.i.d. reference $P_{Y}^{⊗n}$. With $P_{Y}=\mathrm{Unif}\left(Y\right)$ (achieved by choosing $P_{X}$ appropriately), this is a direct test of whether soft covering has succeeded. The annealed Chernoff exponent for this binary hypothesis test equals
  (49) $$\xi \left(R\right)=\max_{0\leq \beta \leq 1}\left[(1-\beta )\log|Y|-\psi (\beta ,R)\right],$$
  where ψ(β,R) is the annealed free energy of Theorem 1. Since both branches of ψ(β,R) are fully characterized by our results, (49) is completely determined. The optimizer $\beta^{🟉}\in \left[0,1\right]$ satisfies $\psi^{\prime }\left(\beta^{🟉},R\right)=-\log\left|Y\right|$, so the phase structure of ψ(β,R) directly governs the behavior of ξ(R): the bulk/sparse phase boundaries produce regime changes in the Chernoff exponent as *R* varies.

With $P_{Y}=\mathrm{Unif}$, two clean consequences follow. First, ξ(R)=0 if and only if R≥I(X;Y): the two distributions are not exponentially distinguishable for R≥I(X;Y); i.e., no test can achieve an exponentially small total error probability exactly at the soft-covering threshold. (Sub-exponential error rates, e.g., polynomial in *n*, are not excluded). Second, (49) is the Legendre–Fenchel transform of ψ(β,R) with respect to β, evaluated at log|Y|; it therefore equals $\frac{1}{n}\log\sum_{y^{n}}P_{Y^{n}|C}{\left(y^{n}\right)}^{\beta^{🟉}}{\left|Y\right|}^{-n(1-\beta^{🟉})}$, which is precisely the Rényi divergence $D_{\alpha }\left(P_{Y^{n}|C}\;∥\;P_{Y}^{⊗n}\right)$ at order $\alpha =\beta^{🟉}\in \left[0,1\right]$. This connects (49) directly to the Rényi resolvability results of Yu and Tan [7], who showed that $D_{\alpha }\left(P_{Y^{n}|C}∥P_{Y}^{⊗n}\right)\to 0$ if and only if R>I(X;Y) for α≤1, while, for α>1, the threshold is $R=I^{b}\left(\alpha \right)$—the bulk condensation boundary of our phase diagram. Our analysis provides the exact *rate* of divergence at every *R* and α and reveals how the two-branch phase structure of ψ(β,R) determines qualitative changes in this rate.

## 6. Conclusions

We developed a statistical-mechanics framework for the output distribution of random codes, centered on the annealed free energy ψ(β,R) of the partition function $Z_{n}\left(\beta \right)=\sum_{y^{n}}{\left[P_{Y^{n}}\left(y^{n}\right)\right]}^{\beta }$ and its two-branch phase structure. This dichotomy between the two branches has concrete operational consequences. For guesswork, the phase structure of ψ(β,R)—both the bulk phase transition at $R=I^{b}\left(\beta \right)$ and the bulk–sparse crossover at $R=R^{🟉}\left(\beta \right)$—translates directly (via Arıkan’s formula [8]) into regime changes in the exponential growth rate of guessing moments $E[G{\left(Y^{n}\right)}^{s}]$. Similar comments apply to hypothesis testing and perhaps other application areas. The annealed free energy adds yet another layer: its phase boundary $R=R^{🟉}\left(\beta \right)$ separates the regime where the ensemble average is dominated by rare atypical codebooks (for example, the sparse branch dominates in Figure 2) from the regime where it reflects the behavior of a typical codebook (the bulk branch dominates in Figure 4). The gap $\psi \left(\beta ,R\right)-\psi_{b}\left(\beta ,R\right)>0$ in the sparse-dominant regime quantifies the extent to which ensemble averages can be misleading—a warning that is particularly relevant when analyzing the security of specific random codes rather than random coding in the mean.

Future research directions include: a rigorous derivation of exponentially tight error exponents for the hypothesis test of Application 4 via the type-class enumeration method of [14], together with a phase-diagram analysis of the Neyman–Pearson exponent tradeoff in the (R,τ) plane; a full treatment of the guesswork regime changes produced by the bulk/sparse phase boundaries; quantitative semantic security bounds in terms of $I^{b}\left(\beta \right)$ and $R^{🟉}\left(\beta \right)$; extension to Markov and mixed sources; connections to mismatched decoding exponents; and a complete treatment of the β<1 regime. Finally, we note that the self-averaging property of $Z_{n}\left(\beta |C\right)$ has been established in Theorem 2: for β≥1 and $R>R^{🟉}\left(\beta \right)$, the bulk branch $\psi_{b}\left(\beta ,R\right)$ coincides with the quenched free energy $\lim_{n\to \infty }\frac{1}{n}E\left\{\log Z_{n}\left(\beta |C\right)\right\}$. Whether the same holds in region C ($I^{b}\left(\beta \right)<R<R^{🟉}\left(\beta \right)$), the condensed phase $R\leq I^{b}\left(\beta \right)$, or for β<1 remains an interesting open problem.

## Figures and Tables

**Table 1:** Summary of the two optimizers.

	$Q_{\beta }^{b}$	$Q_{\beta }^{s}$
Definition	Unconstrained maximizer of $\psi_{b}\left(\beta ,R\right)$ objective (Equation (32))	Gibbs distribution: $Q_{\beta }^{s}\left(y|x\right)\propto {\left[W\left(y|x\right)\right]}^{\beta }$ per letter (Equation (27))
Maximizes	$H_{Q}\left(Y\right)-\beta \left[I_{Q}\left(X;Y\right)+\ell \left(Q_{XY}\right)\right]$	$H_{Q}\left(Y|X\right)-\beta \ell \left(Q_{XY}\right)=F\left(Q_{XY}\right)$
MI at optimum	$I^{b}\left(\beta \right)$	$I^{s}\left(\beta \right)$
Governs	Bulk condensation boundary $R=I^{b}\left(\beta \right)$	Bulk/sparse crossover $R=R^{🟉}\left(\beta \right)$
At β=1	True channel *W*, $I^{b}\left(1\right)=I\left(X;Y\right)$	True channel *W*, $I^{s}\left(1\right)=I\left(X;Y\right)$

## Data Availability

The original contributions presented in this study are included in the article. Further inquiries can be directed to the corresponding author.

## Appendix A. Proof of Theorem 2

Throughout this proof, we define

(A1) $$U_{n}:=\frac{1}{n}\log Z_{n}\left(\beta |C\right).$$

We begin with part (i). To establish the upper bound, observe that, by Jensen’s inequality, $E\left\{U_{n}\right\}\leq \frac{1}{n}\log E\left[Z_{n}\left(\beta |C\right)\right]\to \psi \left(\beta ,R\right)=\psi_{b}\left(\beta ,R\right)$, where the last equality is by the postulate $R\geq R^{🟉}\left(\beta \right)$. It follows that ${lim sup}_{n\to \infty }E\left\{U_{n}\right\}\leq \psi_{b}\left(\beta ,R\right)$. As for the lower bound, fix any joint type $Q_{XY}$ with $Q_{X}=P_{X}$ and $I_{Q}\left(X;Y\right)\leq R$, and let $Q_{Y}$ be its *Y*-marginal. For every output sequence $y^{n}$ of type $Q_{Y}$, each codeword of joint type $Q_{XY}$ with $y^{n}$ contributes exactly $e^{-n\ell (Q_{XY})}$ to $\sum_{m}W^{n}\left(y^{n}|x^{n}\left(m\right)\right)$, so

(A2) $$P_{Y^{n}|C}\left(y^{n}\right)\;\geq \;\frac{N(Q_{XY}|y^{n})}{M}\cdot e^{-n\ell (Q_{XY})},$$

and, since β≥1,

(A3) $$Z_{n}\left(\beta |C\right)\;\geq \;\sum_{\begin{array}{c} y^{n}:\;{\hat{P}}_{y^{n}}=Q_{Y} \end{array}}\;\;{\left(\frac{N(Q_{XY}|y^{n})}{M}\right)}^{\;\beta }e^{-n\beta \ell (Q_{XY})}.$$

As observed before, $N(Q_{XY}|y^{n})$ is binomial with $e^{nR}$ trials and a probability of a single success of the exponential order of $e^{-nI_{Q}\left(X;Y\right)}$, so its mean $\mu_{n}$ is exponentially $e^{n(R-I_{Q}\left(X;Y\right))}\to \infty$. By the Chernoff bound, for any δ∈(0,1),

(A4) $$\mathrm{Pr}\left\{N\left(Q_{XY}|y^{n}\right)\leq \left(1-\delta \right)\mu_{n}\right\}\leq \exp\left(-\frac{\delta^{2}\mu_{n}}{2}\right)=\exp\left(-\frac{\delta^{2}}{2}e^{n[R-I_{Q}\left(X;Y\right)]}\right),$$

which is *doubly* exponentially small (see also Theorem 4.1 of [14] and, in particular, eqs. (D.16) and (D.17) in its proof therein, which establish the doubly exponential decay). A union bound over all joint types $\left\{Q_{XY}\right\}$ and all $|T\left(Q_{Y}\right)|\leq e^{nH_{Q}\left(Y\right)}$ sequences $y^{n}$ gives

(A5) $$\mathrm{Pr}\left\{E_{n}^{c}\right\}\leq {\left(n+1\right)}^{\left|X||Y\right|}\cdot e^{nH_{Q}\left(Y\right)}\cdot \exp\left(-\frac{\delta^{2}}{2}\;e^{n[R-I_{Q}\left(X;Y\right)]}\right)\;⟶\;0,$$

where $E_{n}$ is the event that $N\left(Q_{XY}|y^{n}\right)\geq \left(1-\delta \right)\mu_{n}$ for all types $Q_{XY}$ and all $y^{n}\in T\left(Q_{Y}\right)$ simultaneously. On $E_{n}$, the bound (A3) gives

(A6) $$\begin{array}{cc} Z_{n}\left(\beta |C\right)\; & \geq |T\left(Q_{Y}\right)|\cdot {\left(\frac{\left(1-\delta \right)\mu_{n}}{M}\right)}^{\;\beta }e^{-n\beta \ell (Q_{XY})}\\ \; & \;≐\;{\left(1-\delta \right)}^{\beta }\cdot \exp\;\left(n\left[H_{Q}\left(Y\right)-\beta \left(I_{Q}\left(X;Y\right)+\ell \left(Q_{XY}\right)\right)\right]\right). \end{array}$$

Taking the supremum over all $Q_{XY}$ with $Q_{X}=P_{X}$ and $I_{Q}\left(X;Y\right)\leq R$, we obtain

(A7) $$U_{n}\;\geq \;\psi_{b}\left(\beta ,R\right)+\frac{\log(1-\delta )}{n}\;\mathrm{on}\;E_{n}.$$

Taking expectations of both sides of (A7) and using $U_{n}\geq \left(1-\beta \right)\log\left|Y\right|$ deterministically (since $Z_{n}\left(\beta \right)\geq {\left|Y\right|}^{n(1-\beta )}$ by Jensen):

(A8) $$E\left\{U_{n}\right\}\geq \left(\psi_{b}\left(\beta ,R\right)+\frac{\log(1-\delta )}{n}\right)\mathrm{Pr}\left\{E_{n}\right\}+\left(1-\beta \right)\log\left|Y\right|\cdot \mathrm{Pr}\left\{E_{n}^{c}\right\}.$$

Since $\mathrm{Pr}[E_{n}]\to 1$, we get ${lim inf}_{n\to \infty }E\left\{U_{n}\right\}\geq \psi_{b}\left(\beta ,R\right)$, which, together with

(A9) $$\underset{n\to \infty }{lim sup}E\left\{U_{n}\right\}\leq \psi_{b}\left(\beta ,R\right),$$

implies

(A10) $$\lim_{n\to \infty }E\left\{U_{n}\right\}=\psi_{b}\left(\beta ,R\right),$$

thus completing the proof of part (i).

Proceeding to part (ii), we begin by proving that $\psi_{b}\left(\beta ,R\right)$ is an almost sure upper bound. By Markov’s inequality, for any ε>0,

(A11) $$\mathrm{Pr}\left\{U_{n}\geq \psi_{b}\left(\beta ,R\right)+\varepsilon \right\}\leq \frac{E[Z_{n}\left(\beta |C\right)]}{e^{n(\psi_{b}\left(\beta ,R\right)+\varepsilon )}}≐e^{-n\varepsilon },$$

where we have used the fact that $\frac{1}{n}\log E\left[Z_{n}\left(\beta |C\right)\right]\to \psi_{b}\left(\beta ,R\right)$, which holds because $\psi \left(\beta ,R\right)=\psi_{b}\left(\beta ,R\right)$ for $R>R^{🟉}\left(\beta \right)$. Since $\sum_{n}e^{-n\varepsilon }<\infty$, the Borel–Cantelli lemma yields $U_{n}\leq \psi_{b}\left(\beta ,R\right)+\varepsilon$ for every ε>0 eventually a.s., or, equivalently, ${lim sup}_{n\to \infty }U_{n}\leq \psi_{b}\left(\beta ,R\right)$ a.s. For the compatible almost sure lower bound, note that we have already proved in (A7) that, whenever $E_{n}$ occurs, $U_{n}\geq \psi_{b}\left(\beta ,R\right)+\frac{\log(1-\delta )}{n}$, which means that $E_{n}\subseteq F_{n}:=\left\{U_{n}\geq \psi_{b}\left(\beta ,R\right)+\frac{\log(1-\delta )}{n}\right\}$, or, equivalently, $F_{n}^{c}\subseteq E_{n}^{c}$. Thus,

(A12) $$\begin{array}{c} \sum_{n=1}^{\infty }Pr\left\{U_{n}<\psi_{b}\left(\beta ,R\right)+\frac{\log(1-\delta )}{n}\right\}=\sum_{n=1}^{\infty }\mathrm{Pr}\left\{F_{n}^{c}\right\}\leq \sum_{n=1}^{\infty }\mathrm{Pr}\left\{E_{n}^{c}\right\}<\infty \end{array}$$

where the summability of $\mathrm{Pr}\{E_{n}^{c}\}$ is due to the fact that each term is bounded by a doubly exponentially small quantity. By the Borel–Cantelli lemma, $U_{n}-\frac{\log(1-\delta )}{n}\geq \psi_{b}\left(\beta ,R\right)$ eventually a.s., or, equivalently, ${lim inf}_{n\to \infty }U_{n}\geq \psi_{b}\left(\beta ,R\right)$ a.s., which, together with the matching upper bound, yields $\lim_{n\to \infty }U_{n}=\psi_{b}\left(\beta ,R\right)$ a.s., completing the proof.

## References

[1] Han T.S., Verdú S. (1993). Approximation theory of output statistics. IEEE Trans. Inf. Theory.

[2] Cuff P. Soft covering with high probability. Proceedings of the 2016 IEEE International Symposium on Information Theory (ISIT).

[3] Hayashi M. (2006). General nonasymptotic and asymptotic formulas in channel resolvability and identification capacity and their application to wire-tap channel. IEEE Trans. Inf. Theory.

[4] Hou J., Kramer G. Effective secrecy: Reliability, confusion and stealth. Proceedings of the 2014 IEEE International Symposium on Information Theory.

[5] Mézard M., Montanari A. (2009). Information, Physics, and Computation.

[6] Merhav N. (2010). Statistical physics and information theory. Found. Trends Commun. Inf. Theory.

[7] Yu L., Tan V.Y.-F. (2019). Rényi resolvability and its applications to the wiretap channel. IEEE Trans. Inf. Theory.

[8] Arıkan E. (1996). An inequality on guessing and its application to sequential decoding. IEEE Trans. Inf. Theory.

[9] Blahut R.E. (1974). Hypothesis testing and information theory. IEEE Trans. Inf. Theory.

[10] Sourlas N. (1989). Spin-glass models as error-correcting codes. Nature.

[11] Montanari A. (2001). Turbo codes: The phase transition. Eur. Phys. J. B.

[12] Csiszár I., Körner J. (2011). Information Theory: Coding Theorems for Discrete Memoryless Systems.

[13] Csiszár I. (1998). The method of types. IEEE Trans. Inf. Theory.

[14] Merhav N., Weinberger N. (2025). A toolbox for refined information-theoretic analyses with applications. Found. Trends Commun. Inf. Theory.

